# Specific amino acids but not total protein attenuate postpartum weight gain among Hispanic women from Southern California

**DOI:** 10.1002/fsn3.2085

**Published:** 2021-02-13

**Authors:** Laura E. Wild, Tanya L. Alderete, Noopur C. Naik, William B. Patterson, Paige K. Berger, Roshonda B. Jones, Jasmine F. Plows, Michael I. Goran

**Affiliations:** ^1^ Department of Integrative Physiology University of Colorado Boulder Boulder CO USA; ^2^ Department of Pediatrics The Saban Research Institute Children's Hospital Los Angeles University of Southern California Los Angeles CA USA

**Keywords:** amino acids, diet, Hispanics, maternal health, postpartum weight, postpartum women, protein

## Abstract

There is a high prevalence of obesity and type 2 diabetes in the United States, particularly among Hispanic women, which may be partly explained by failure to lose gestational weight during the postpartum period. Previous work indicates that protein and amino acids may protect against weight gain; therefore, this study examined the impact of dietary protein and amino acid intake on changes in postpartum weight and the percent of women meeting the Estimated Average Requirement (EAR) for these dietary variables among Hispanic women from the Southern California Mother's Milk Study (*n* = 99). Multivariable linear regression analysis was used to examine the associations between protein and amino acid intake with change in weight after adjusting for maternal age, height, and energy intake. Women's weight increased from prepregnancy to 1‐month and 6‐months postpartum (71.1 ± 14.6 vs. 73.1 ± 13.1 vs. 74.5 ± 14.6 kg, *p* < .0001). Although dietary protein was not associated with weight change (*β* = −1.09; *p* = .13), phenylalanine (*β* = −1.46; *p* = .04), tryptophan (*β* = −1.71; *p* = .009), valine (*β* = −1.34; *p* = .04), isoleucine (*β* = −1.26; *p* = .045), and cysteine (*β* = −1.52; *p* = .02) intake were inversely associated with weight change. Additionally, fewer women met the EAR values for cysteine (11.1%), phenylalanine (60.6%), and methionine (69.7%), whereas most women met the EAR values for tryptophan (92.9%), valine (96.0%), and isoleucine (94.9%). Study results indicate that several essential and conditionally essential amino acids were associated with postpartum weight loss, with a significant portion of women not meeting recommended intake levels for some of these amino acids. These results highlight the importance of postpartum maternal diet as a potential modifiable risk factor.

## Graphical abstract

Among 99 Hispanic women from the Southern California Mother's Milk Study, dietary protein was not associated with postpartum weight change; however, several amino acids (i.e., phenylalanine, tryptophan, valine, isoleucine, and cysteine) were inversely associated with change in weight from 1‐ to 6‐months postpartum. Additionally, less than 50% of women were consuming the recommended amounts of dietary protein, tyrosine, and cysteine. These results suggest that dietary protein and amino acids should be considered when developing obesity prevention strategies for Hispanic women during the postpartum period.

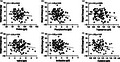

## 1 INTRODUCTION

In the United States, 71.6% of adults have overweight or obesity (CDC, 2018) with a significantly higher prevalence among Hispanics compared to non‐Hispanic whites (Hales et al., 2017; Ogden et al., 2014). These disparities are especially apparent among women where 35.8% of Hispanic women of childbearing age have obesity compared to only 27.8% of non‐Hispanic white women (Ogden et al., 2014). Further, up to 50% of Hispanic women gain excessive weight during pregnancy (Brawarsky et al., 2005; Walker et al., 2009), and weight gained during this time frame often increases risk for postpartum weight retention as well as subsequent maternal obesity. Therefore, excess gestational weight gain coupled with postpartum weight retention may increase the risk of overweight/obesity and type 2 diabetes among Hispanics (Berggren et al., 2016; Ehrlich et al., 2014; Endres et al., 2015; Gunderson, 2009; Martin et al., 2017), contributing to future obesity in their offspring. Despite this, there are limited studies examining the impact of maternal diet on postpartum weight change, particularly among vulnerable populations such as Hispanic women.

Studies suggest that increased protein consumption (Campos‐Nonato et al., 2017; Weigle et al., 2005), diets rich in branched chain amino acids (i.e., leucine, isoleucine, and valine) (Qin et al., 2011), dietary cysteine (McGavigan et al., 2015), and dietary tryptophan (Cavaliere & Medeiros‐Neto, 1997; Virtue et al., 2019) protect against weight gain by increasing energy expenditure (Leidy et al., 2015; Westerterp‐Plantenga et al., 2011) as well as increasing satiety and decreasing total energy intake (Dong et al., 2013; Leidy et al., 2015; Westerterp‐Plantenga et al., 2011). Additionally, adequate protein intake during and after pregnancy is important to maintain maternal health and for milk production during lactation (Kalhan, 2000; King, 2000; Manjarin et al., 2014). At the same time, nutrient inadequacy has been shown to play an important role in the development of overweight and obesity (Kaidar‐Person et al., 2008; Via, 2012). Thus, dietary protein and amino acids have the potential to foster postpartum weight loss, yet many Hispanic women may not be consuming the recommended daily intake of these dietary factors during the postpartum period.

To our knowledge, no studies have examined the impact of protein and amino acids on postpartum weight change among Hispanic women, a population already at increased risk for obesity and type 2 diabetes (Mercader & Florez, 2017; Yracheta et al., 2015). Therefore, the primary aim of this study was to determine whether total protein or individual amino acids in the diet of Hispanic, lactating women were associated with changes in weight from 1‐ to 6‐months postpartum. As a secondary aim, we sought to determine whether participants met the Estimated Average Requirement (EAR) values for lactating women for total protein, essential amino acids (i.e., histidine, isoleucine, leucine, lysine, methionine, phenylalanine, threonine, tryptophan, and valine), nonessential amino acids (i.e., alanine, aspartic acid, glutamic acid, and serine), and conditionally essential amino acids (e.g., proline, cysteine, tyrosine, and glycine).

## 2 METHODS

### 2.1 Study participants

The 99 participants in this study were recruited between 2016 and 2019 for the Southern California Mother's Milk Study (NIH R01 DK110793). The Mother's Milk Study is an ongoing study that examines breast milk factors and the gut microbiota during the first two years of infant life (Berger et al., 2019). Mother–infant pairs are being recruited from maternity clinics affiliated with the University of Southern California in Los Angeles County. Inclusion criteria include self‐identification of Hispanic ethnicity; greater than 18 years old at the time of delivery; a healthy, term, singleton birth; within 1‐month postpartum; and intention to breastfeed for at least 3 months postpartum. Participants taking medications or with any medical conditions impacting metabolism, nutritional status, and physical or mental health were excluded from the study. Participants using tobacco (i.e., >1 cigarette per week) or recreational drugs as well as those that had a clinical diagnosis of fetal abnormalities were excluded from the Mother's Milk Study. To date, 217 participants have been enrolled in the Mother's Milk Study and 104 had available dietary data at 1‐ and 6‐months postpartum at the time of the current study. Of these 104 participants, one outlier was removed due to excessive weight loss, which was more than 11 standard deviations above the mean. Additionally, although beyond the scope of the current study, we have found that added sugar may also impact postpartum weight change in this cohort of Hispanic women. Therefore, four additional outliers were removed due to high intake of dietary sugar (defined as greater than two standard deviations above the mean) (Figure S1). Written informed consent was obtained from participants prior to testing, and the Institutional Review Boards (IRB) at the University of Southern California (USC) and the Children's Hospital Los Angeles (CHLA) approved this study.

### 2.2 Study visits

For the purpose of the study, participants completed one clinical visit at 1‐month postpartum, and another at 6‐months postpartum. At each of these visits, information regarding family and maternal health was collected. Additionally, maternal weight (kg) was measured using an electronic scale and standing height was measured using a stadiometer (m) in order to calculate maternal body mass index (BMI, kg/m^2^). Maternal prepregnancy weight, age at delivery, delivery mode, and infant sex were collected at the first clinical visit. Maternal BMI was used to classify mothers as normal weight, overweight, and obese based on the Center for Disease Control (CDC) and Prevention standards (CDC, 2019).

### 2.3 Dietary intake and physical activity

Two nonconsecutive 24‐hr diet recalls were performed at each 1‐ and 6‐months postpartum visit, which were used to represent average maternal dietary intake. Dietary recalls were used to assess dietary information including dietary supplements, total protein, essential amino acids (i.e., histidine, isoleucine, leucine, lysine, methionine, phenylalanine, threonine, tryptophan, and valine), nonessential amino acids (i.e., alanine, aspartic acid, glutamic acid, and serine), and conditionally essential amino acids (e.g., cysteine, proline, tyrosine, glycine, and arginine). Briefly, a trained and bilingual registered dietitian assessed maternal daily intake of dietary factors separately at 1‐ and 6‐months postpartum. At each time point, recalls included 1 weekday and 1 weekend day using the 5‐step, multi‐pass method (Steinfeldt et al., 2013). The first recall was performed in person at our laboratory with the use of food models, portion booklets, or serving containers to assist in estimating serving sizes. The remaining recalls were conducted by telephone. A trained research assistant coded and analyzed all dietary recall data, which was entered into the Nutrition Data System for Research (NDSR) software (version 2018). In NDSR, total protein included meat, fish, poultry, eggs, dairy products, cereals, legumes and nuts, vegetables, and fruits. In addition, amino acid values included both protein‐bound and free forms that are expressed in grams (g). Dietary cystine also includes cysteine; for this reason, cystine was examined as cysteine in the current analysis. To assess nutrient inadequacies, we utilized the Estimate Average Requirement (EAR) values developed by the Food and Nutrition Board of the Institute of Medicine for lactating women 19–50 years old (NIH, 2019). Since EAR values were listed in grams of intake per kilogram of bodyweight, EAR intakes for lactating women (IOM, 2005) were calculated for each individual woman based on her average maternal weight from 1‐ to 6‐months postpartum. Physical activity was assessed using the metabolic equivalent of task (MET) score, which was obtained from a 3‐day physical activity recall (PAR) administered at the 1‐ and 6‐months study visits. In order to capture physical activity during the study period, the mean of the average 3‐day score from both time points was calculated (Han & Dinger, 2013).

### 2.4 Statistical analysis

Means and standard deviations (mean ± *SD*) are reported for continuous variables, and frequencies and percentages are reported for categorical variables. Maternal dietary information was captured using the average of dietary recalls performed at 1‐ and 6‐months postpartum. At 1‐month, all women reported that they were breastfeeding, and at 6‐months, 80.8% reported that they were breastfeeding (*n* = 80). Therefore, the EAR values examined were specific to lactating women (when available) to determine the proportion of women meeting these guidelines. Differences in maternal weight were determined using one‐way repeated measures analysis of variance (ANOVA). Following this, post hoc tests were used to determine statistically significant differences between each time point (e.g., prepregnancy, 1‐and 6‐months) using a Bonferroni for correction for multiple testing. We then examined whether protein and amino acid intakes were associated with postpartum weight change. For these analyses, multiple linear regression models were used to examine the associations between protein and amino acids and change in maternal weight from 1‐ to 6‐months postpartum. These models controlled for maternal age at 1‐month postpartum, maternal height, and average energy intake from 1‐ to 6‐months postpartum as a priori covariates. Results were largely unchanged after additionally adjusting for prepregnancy maternal weight, change in breast feeding status, or physical activity (Table S1). Amino acids that were found to be inversely associated with postpartum weight change remained significant after additionally adjusting for total protein; however, effect estimates were unreliable as dietary protein was strongly correlated with amino acids. It was not possible to construct multivariable models that included more than one amino acid due to high correlations among these variables (*r* > .9) (Table S2). In order to better interpret the effect sizes, we scaled betas by a one‐standard deviation (*SD*) increase in protein (*SD* = 17.37 g), cysteine (*SD* = 0.24 g), proline (*SD* = 1.09 g), phenylalanine (*SD* = 0.76 g), tryptophan (*SD* = 0.21 g), valine (*SD* = 0.91 g), isoleucine (*SD* = 0.83 g), arginine (*SD* = 1.06 g), methionine (*SD* = 0.46 g), leucine (*SD* = 1.43 g), lysine (*SD* = 1.4 g), histidine (*SD* = 0.52 g), threonine (*SD* = 0.73 g), alanine (*SD* = 0.94 g), aspartic acid (*SD* = 1.61 g), glycine (*SD* = 0.80 g), glutamic acid (*SD* = 3.17 g), serine (*SD* = 0.81 g), and tyrosine (*SD* = 0.64 g), which better represented intakes observed in our population. Statistical significance was based on a two‐sided *p* < .05. All analyses were performed in RStudio (Version 1.1.456).

## 3 RESULTS

This study included 99 participants from the Mother's Milk Study. At 1‐month postpartum, the participants had a mean age of 29 years (range 20–45 years). The average dietary protein and amino acid intake values are shown in Table 1. We found that women were overweight prior to the pregnancy period after calculating their average BMI based on self‐reported weight (kg) and height (m) (28.02 ± 5.63 kg/m^2^). From prepregnancy to 6‐months postpartum, women increased their body weight (*p* < .0001). For example, women increased their weight by an average of 2.07 kg (*SD* = 6.95 kg, *p* = .01) from prepregnancy to 1‐month postpartum, resulting in an average increase of 1.78 kg/m^2^ BMI units (29.8 ± 4.8 kg/m^2^). From 1‐month to 6‐months, 61.6% of women gained weight (*n* = 61) and 38.4% lost weight (*n* = 38); however, the average weight gain among all women was 1.38 kg (*SD* = 4.22, *p* = .005). Thus, at 6‐months postpartum, the average maternal weight was 74.5 kg (*SD* = 14.6 kg), resulting in an average increase of 0.5 kg/m^2^ BMI units (30.3 ± 5.5 kg/m^2^) from the 1‐month postpartum visit.

**TABLE 1:** Average dietary protein and amino acid intake from recalls performed at 1‐ and 6‐months postpartum

Nutrient	Mean ± *SD*
Dietary intake	
Energy intake (kcal)	1,665.73 ± 374.99
Protein (g/d)	76.24 ± 17.92
Percent protein (%kcal/d)	18.85 ± 3.28
Conditionally essential amino acids	
Cysteine (g/d)	1.02 ± 0.25
Arginine (g/d)	4.19 ± 1.09
Glycine (g/d)	3.20 ± 0.83
Proline (g/d)	4.61 ± 1.11
Tyrosine (g/d)	2.62 ± 0.66
Essential amino acids	
Phenylalanine (g/d)	3.33 ± 0.78
Tryptophan (g/d)	0.87 ± 0.21
Valine (g/d)	3.83 ± 0.93
Isoleucine (g/d)	3.43 ± 0.85
Methionine (g/d)	1.78 ± 0.47
Leucine (g/d)	6.05 ± 1.47
Lysine (g/d)	5.28 ± 1.45
Histidine (g/d)	2.13 ± 0.53
Threonine (g/d)	3.01 ± 0.75
Nonessential amino acids	
Alanine (g/d)	3.81 ± 0.97
Aspartic acid (g/d)	6.79 ± 1.67
Glutamic acid (g/d)	14.09 ± 3.25
Serine (g/d)	3.47 ± 0.83

Note

This table shows mean ± standard deviation (*SD*) for the 99 Hispanic women included in this study. Dietary intake was determined from 24‐hr dietary recalls performed at 1‐ and 6‐months postpartum. The table shows each dietary factor, which was derived from the average of the 1‐ and 6‐months recalls.

### 3.1 Amino acids were inversely associated with postpartum weight change

**FIGURE 1:**
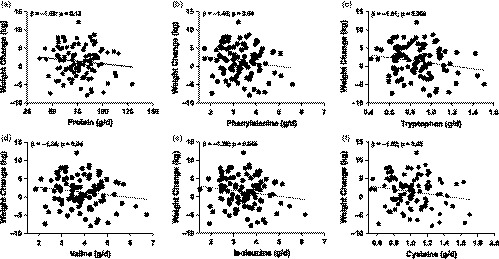
Dietary amino acids were associated with weight loss in Hispanic, postpartum women 1‐ and 6‐months postpartum. Dietary amino acid intake was associated with maternal weight loss from 1‐ to 6‐months postpartum in 99 women. Average dietary intake was determined from 24‐hr dietary recalls performed at 1‐ and 6‐months postpartum. Unadjusted values for change in maternal weight from 1‐ to 6‐months compared with protein are shown. Multiple linear regression was performed to obtain the parameter estimates (*β*) after adjustment for mother's age at 1‐month postpartum, height, and average energy intake (kcal). Reported betas (*β*) are shown for a one‐standard deviation increase in (a) protein (*SD* = 17.37 g/d), (b) phenylalanine (*SD* = 0.76 g/d), (c) tryptophan (*SD* = 0.21 g/d), (d) valine (*SD* = 0.91 g), (e) isoleucine (*SD* = 0.83 g/d), and (f) cysteine (*SD* = 0.24 g/d)

Animal protein (e.g., poultry, beef, and fish) and milk were the largest sources of protein and amino acid intake among Hispanic postpartum women. Although protein was not found to be significantly associated with postpartum weight change (*β* = −1.09; *p* = .13), our results indicate that higher consumption of specific amino acids attenuated the average weight gain that was observed among Hispanic women. As such, intake of four essential amino acids (phenylalanine, tryptophan, valine, and isoleucine) were each inversely associated with change in maternal weight from 1‐ to 6‐months postpartum (Table 2 and Figure 1). For example, a one‐standard deviation increase of phenylalanine intake (0.76 g) was associated with a 1.46 kg weight loss (*p* = .04). Also, a one‐standard deviation increase in tryptophan (0.21 g) and valine (0.91 g) was associated with 1.71 kg (*p* = .009) and 1.34 kg decrease (*p* = .04) in maternal weight, respectively. For isoleucine, a one‐standard deviation increase (0.83 g) was associated with a 1.26 kg decrease in maternal weight (*p* = .045). Of the conditionally essential amino acids, only cysteine was associated with a decrease in postpartum weight. Specifically, a one‐standard deviation increase in cysteine intake (0.24 g) was associated with a 1.52 kg weight loss (*p* = .02). Lastly, nonessential amino acids and protein were not significantly associated with change in maternal weight.

**TABLE 2:** Dietary protein and amino acids were associated with postpartum weight from 1‐ and 6‐months postpartum

Nutrient	*β*	*p*‐value
Protein (g/d)	−1.09	.13
Conditionally essential amino acids		
Cysteine (g/d)	−1.52	**.02**
Arginine (g/d)	−0.74	.22
Glycine (g/d)	−0.61	.32
Proline (g/d)	−0.99	.23
Tyrosine (g/d)	−1.28	.06
Essential amino acids		
Phenylalanine (g/d)	−1.46	**.04**
Tryptophan (g/d)	−1.71	**.009**
Valine (g/d)	−1.34	**.04**
Isoleucine (g/d)	−1.26	**.045**
Methionine (g/d)	−0.84	.18
Leucine (g/d)	−1.03	.13
Lysine (g/d)	−0.80	.18
Histidine (g/d)	−0.98	.12
Threonine (g/d)	−1.13	.07
Nonessential amino acids		
Alanine (g/d)	−0.67	.27
Aspartic Acid (g/d)	−1.14	.07
Glutamic Acid (g/d)	−1.33	.10
Serine (g/d)	−1.18	.09

Note

Average dietary intake was determined from 24‐hr dietary recalls performed at 1‐ and 6‐months postpartum. Dietary protein and amino acids found to be significantly associated with average weight change from 1‐ to 6‐months postpartum (*n* = 99) are shown in bold. Model adjusted for age at 1‐month postpartum, height, and total energy intake (kcal). Reported betas (*β*) are shown for a one‐standard deviation (*SD*) increase in protein (*SD* = 17.37 g), cysteine (*SD* = 0.24 g), proline (*SD* = 1.09 g), phenylalanine (*SD* = 0.76 g), tryptophan (*SD* = 0.21 g), valine (*SD* = 0.91 g), isoleucine (*SD* = 0.83 g), arginine (*SD* = 1.06 g), methionine (*SD* = 0.46 g), leucine (*SD* = 1.43 g), lysine (*SD* = 1.4 g), histidine (*SD* = 0.52 g), threonine (*SD* = 0.73 g), alanine (*SD* = 0.94 g), aspartic acid (*SD* = 1.61 g), glycine (*SD* = 0.80 g), glutamic acid (*SD* = 3.17 g), serine (*SD* = 0.81 g), and tyrosine (*SD* = 0.64 g).

These value represent significant *p* values.

### 3.2 Hispanic women had inadequate intake of total protein and some of the amino acids that were negatively associated with postpartum weight change

As a secondary aim, we sought to determine whether postpartum Hispanic women were meeting the Estimated Average Requirement (EAR) values for protein, essential amino acids, nonessential amino acids, and conditionally essential amino acids (e.g., proline, cysteine). Overall, postpartum Hispanic women were largely inadequate in total protein intake, four essential amino acids, and one conditionally essential amino acid that was associated with postpartum weight loss (Table 3). For example, only 49.5% of women consumed the estimated daily requirement amount for total protein. Additionally, only 11.1% of women were meeting the calculated EAR values for cysteine and 60.6% of women for phenylalanine. Although dietary tryptophan was inversely associated with postpartum weight change, most women (92.9%) met the calculated EAR for this amino acid. Two additional essential amino acids were also inversely associated with postpartum weight change, yet most women met the EAR values for valine (96.0%) and isoleucine (94.9%). Lastly, although not associated with postpartum change in weight, women were largely not consuming recommended amounts of methionine and tyrosine, with only 69.7% and 32.3% of women meeting these EARs, respectively.

**TABLE 3:** Percent of postpartum Hispanic women meeting protein and amino estimated average requirement (EAR) values during lactation

Nutrient	EAR^a^	Mean ± *SD*	% meeting EAR
Protein (g/d)	1.05	76.24 ± 17.92	49.5%
Conditionally essential amino acids			
Cysteine (g/d)	0.021	1.02 ± 0.25	11.1%
Tyrosine (g/d)	0.041	2.62 ± 0.66	32.3%
Essential amino acids			
Phenylalanine (g/d)	0.041	3.33 ± 0.78	60.6%
Tryptophan (g/d)	0.007	0.87 ± 0.21	92.9%
Valine (g/d)	0.028	3.83 ± 0.93	96.0%
Isoleucine (g/d)	0.024	3.43 ± 0.85	94.9%
Methionine (g/d)	0.021	1.78 ± 0.47	69.7%
Leucine (g/d)	0.050	6.05 ± 1.47	90.9%
Lysine (g/d)	0.042	5.28 ± 1.45	89.9%
Histidine (g/d)	0.015	2.13 ± 0.53	94.9%
Threonine (g/d)	0.024	3.01 ± 0.75	94.9%

Note

This tables show mean ± standard deviation (*SD*) for those amino acids with an estimated average requirement (EAR) value (g/kg/d). Average dietary intake was determined from 24‐hr dietary recalls performed at 1‐ and 6‐months postpartum. Percent (%) meeting EAR was calculated based on each women's individual average maternal weight from 1‐ to 6‐months postpartum.

^a^ EAR (g/kg/d) comes from the National Institutes of Health for lactating women 19–50 years.

## 4 DISCUSSION

Adequate dietary protein intake is essential for women of childbearing age, especially during the postpartum period due to the need to support lactation (Kalhan, 2000; King, 2000; Manjarin et al., 2014). Additionally, dietary protein has been shown to aid in weight loss and weight management (Dong et al., 2013; Leidy et al., 2015; Morenga & Mann, 2012; Westerterp‐Plantenga et al., 2011) by increasing energy expenditure (Leidy et al., 2015; Westerterp‐Plantenga et al., 2011), as well as increasing satiety and decreasing total energy intake (Dong et al., 2013; Leidy et al., 2015; Westerterp‐Plantenga et al., 2011). Beyond total protein, specific amino acids such as histidine, tryptophan, tyrosine, and cysteine have been shown to increase satiety (Kinsey‐Jones et al., 2017; Ayaso et al., 2014; Heeley & Blouet, 2016; Jordi et al., 2013; McGavigan et al., 2015; Michelle, 2015; Tian et al., 2019) and could be protective for healthy weight. Therefore, the aim of this study was to determine whether protein and amino acids were associated with postpartum weight change and whether postpartum Hispanic women were meeting the EAR values for these dietary factors. Results from this study found that five amino acids (i.e., phenylalanine, tryptophan, valine, isoleucine, and cysteine) were inversely associated with maternal weight change from 1‐ to 6‐months postpartum. Further, results show that during the postpartum period, under 50% of Hispanic women were consuming recommended amounts of dietary protein, which could explain why they were also not meeting requirements for individual amino acids tyrosine and cysteine. To our knowledge, this is the first study to indicate that essential and conditionally essential amino acids are associated with postpartum weight loss with potentially important inadequacies in some of these amino acids.

In the current study, phenylalanine was negatively associated with postpartum weight change and 60.6% of Hispanic women consumed the EAR for this essential amino acid. Dietary phenylalanine may aid in weight loss through its effects on catabolic processes, such as lipolysis and beta‐oxidation. For example, one study found that glucagon levels rapidly increased after phenylalanine ingestion (Nuttall et al., 2006), which stimulates and increases fatty acid beta‐oxidation in the liver (Rui, 2014). Another study also found that phenylalanine supplementation, in conjunction with exercise, decreased subcutaneous fat by increasing fat mobilization and oxidation through increased pancreatic alpha‐cell activity (Ueda et al., 2018). In addition, two branched chain amino acids (i.e., valine and isoleucine) were associated with postpartum weight loss among Hispanic women; however, most women met the EAR values for valine (96.0%) and isoleucine (94.9%). Greater intake of valine and isoleucine could improve body composition (Martens & Westerterp‐Plantenga, 2014), thereby aiding in weight loss through an increased basal metabolic rate (Connolly et al., 1999). For example, valine and isoleucine have been shown to stimulate muscle protein synthesis via increased mTORC1 signaling (Jackman et al., 2017; Moberg et al., 2016). Supporting this hypothesis, diet‐induced obese mice fed isoleucine in drinking water gained less weight and had a lower accumulation of white adipose tissue compared to the control group (Nishimura et al., 2010).

In addition to valine and isoleucine, dietary cysteine was negatively associated with weight change from 1‐ to 6‐months postpartum; however, only 11.1% of women meet the EAR values for this conditionally essential amino acid. Cysteine is a sulfur‐containing amino acid that is largely found in meats and fish (Nimni et al., 2007), and cystine is formed from the dimerization of two cysteine molecules (Olsen et al., 2018). It is classified as a conditionally essential amino acid, which means it is normally synthesized by the body but can become essential during times of stress and illness (Riedijk et al., 2007), which may include obesity or lactation. To date, studies investigating the associations between dietary cysteine and/or cystine with weight loss have shown differing results. For example, high compared to low dietary cystine has been shown to promote adiposity in rodents (Elshorbagy et al., 2012), yet dietary l‐cysteine has been shown to suppress ghrelin and reduce appetite in rodents and humans (McGavigan et al., 2015). Given these mixed findings, future work is needed in order to understand how dietary cysteine may contribute to postpartum weight loss. Lastly, we observed that 92.9% of the women were meeting the EAR for dietary tryptophan, which was also inversely associated with postpartum change in weight. Therefore, even though most women were meeting the EAR for tryptophan, higher levels of consumption may be protective of further weight gain. For example, some (Hrboticky et al., 1985; Morris et al., 1987), but not all (Frank & Menden, 1994), studies suggest that tryptophan may play an important role in reducing food intake by stimulating gut hormones relevant to appetite suppression and glycemic control, including cholecystokinin (CCK) and glucagon (Geary et al., 1992; Steinert et al., 2014). In addition, tryptophan is an essential amino acid precursor for the production of serotonin, which can suppress appetite by acting on pro‐opiomelanocortin (POMC) and/or cocaine‐ and amphetamine‐regulated transcript (CART) neurons (Kim et al., 2014).

This is the first study to examine the associations between dietary protein and amino acids with postpartum weight change among Hispanic women while also characterizing intakes with respect to the EAR values during a vulnerable time period for women's health. Despite these strengths, this study is limited by the exclusion of other racial/ethnic groups, which precludes generalizability. However, results from this study are especially important since Hispanic women are at increased risk for obesity. This study used multiple 24‐hr diet recalls at 1‐ and 6‐months postpartum to estimate an average maternal intake of dietary protein and amino acids. Although each recall was performed by trained staff, and we employed the multi‐pass method, dietary recalls can be subject to bias via underreporting of less healthful foods and are reliant upon the memory of participants (Wrieden et al., 2003). Additionally, the use of dietary recalls at only 1‐ and 6‐months postpartum may not have fully captured maternal dietary patterns during this dynamic time period. Future studies should include additional dietary assessments among a diverse sample of postpartum women. In addition, this study is limited by a relatively small sample size; however, detailed information pertinent to important covariates was available which enabled the adjustment for relevant confounders in several model iterations. Nonetheless, future studies should include a larger sample of women. Lastly, although this study focused specifically on protein and amino acid intake during the postpartum period, in a separate analysis, we have also found that added sugar increased risk for postpartum weight gain whereas dietary soluble fiber was protective in this cohort of Hispanic women.

Results from this study indicate that Hispanic women are not consuming adequate amounts of dietary protein and some amino acids during the lactation period. Notably, results from this study suggest that increased consumption of phenylalanine, tryptophan, valine, isoleucine, and cysteine may reduce the risk for obesity by attenuating weight gain among Hispanic women. For example, in the current study, Hispanic women consuming dietary phenylalanine at the 75th (3.90 g) compared to the 25th (2.73 g) percentile would be predicted to gain 2.20 fewer kilograms during the postpartum period. Women consuming tryptophan at the 75th percentile (0.99 g) compared to the 25th percentile (0.69 g) would be predicted to gain 2.5 fewer kilograms from 1‐ to 6‐months postpartum. For valine and isoleucine, those consuming levels at the 75th percentile compared to the 25th percentile (4.43 g vs. 3.06 g and 4.03 g vs. 2.82 g, respectively) would be predicted to gain 2.0 and 1.8 fewer kilograms from 1‐ to 6‐months postpartum, respectively. Finally, Hispanic women consuming dietary cysteine at the 75th (1.18 g) compared to the 25th (0.82 g) percentile would be predicted to gain 2.2 fewer kilograms during the postpartum period. These findings suggest that dietary protein and amino acids should be considered when developing obesity prevention strategies for Hispanic women during the postpartum period. However, future studies are needed in order to fully characterize the impact of dietary protein and amino acids on maternal body weight.

## CONFLICT OF INTEREST

MIG receives book royalties from Penguin/Avery and is a scientific consultant for YUMI foods.

## Funding information

National Institute of Diabetes and Digestive and Kidney Diseases (NIH R01DK110793), the Gerber Foundation, and the National Institute of Environmental Health Sciences (NIH R00ES027853).

## Supporting information

Fig S1

Table S1

Table S2

## Data Availability

The data that support the findings of this study are available on request from the corresponding author. The data are not publicly available due to privacy or ethical restrictions.
