# Supplementary material for: Specific amino acids but not total protein attenuate postpartum weight gain among Hispanic women from Southern California
Source: Food Sci Nutr. 2021 Feb 13;9(4):1842–50. doi: 10.1002/fsn3.2085 (PMC8020954; doi:10.1002/fsn3.2085)
Supplement: Supplementary file 1 — Fig S1 [file FSN3-9-1842-s001.pptx]

## Slide 1
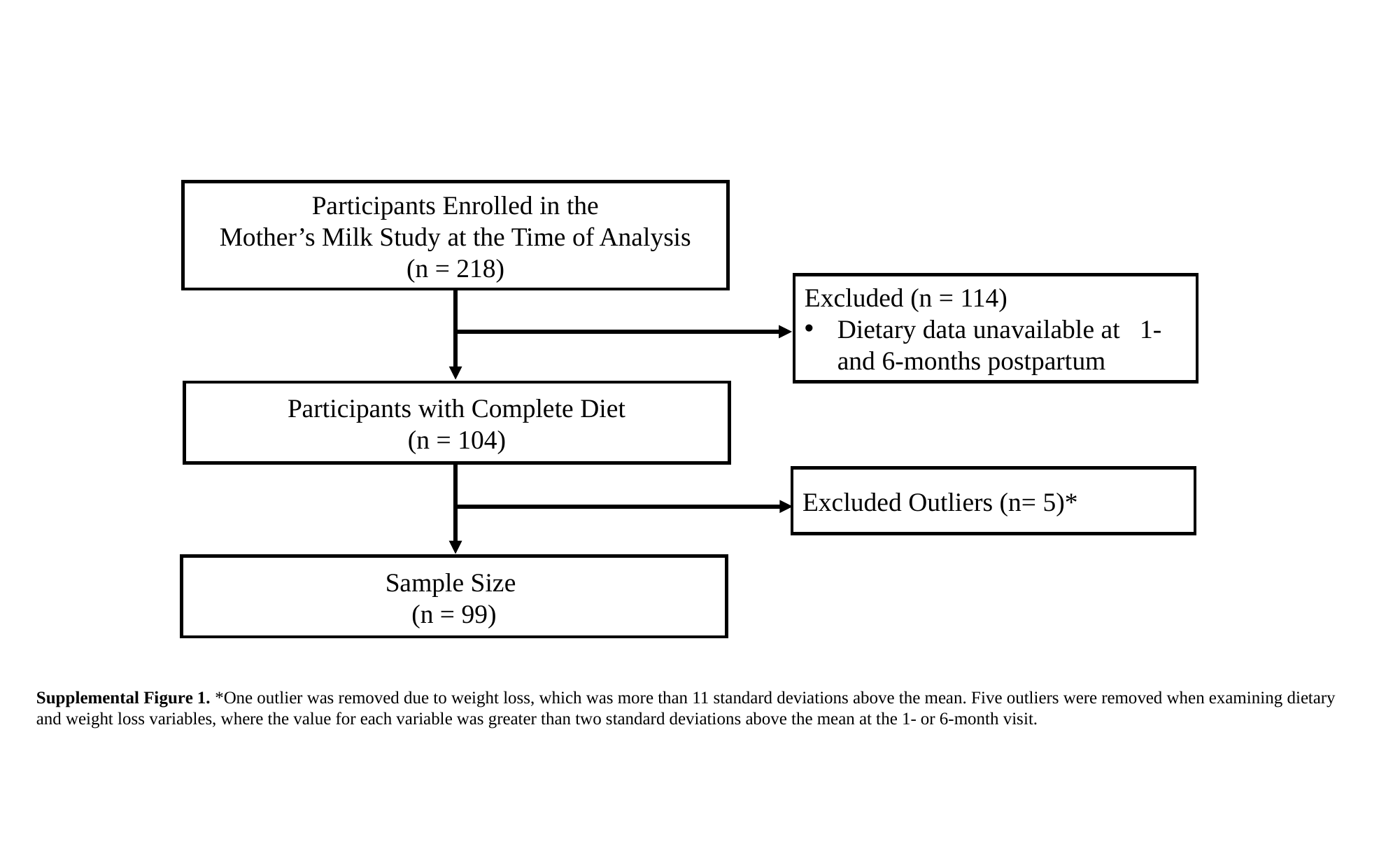

Participants Enrolled in the
Mother’s Milk Study at the Time of Analysis
(n = 218)
Excluded (n = 114)
Dietary data unavailable at 1- and 6-months postpartum
Participants with Complete Diet
(n = 104)
Excluded Outliers (n= 5)*
Sample Size
(n = 99)
Supplemental Figure 1. *One outlier was removed due to weight loss, which was more than 11 standard deviations above the mean. Five outliers were removed when examining dietary and weight loss variables, where the value for each variable was greater than two standard deviations above the mean at the 1- or 6-month visit.
