# Supplementary material for: Specific amino acids but not total protein attenuate postpartum weight gain among Hispanic women from Southern California
Source: Food Sci Nutr. 2021 Feb 13;9(4):1842–50. doi: 10.1002/fsn3.2085 (PMC8020954; doi:10.1002/fsn3.2085)
Supplement: Supplementary file 2 — Table S1 [file FSN3-9-1842-s002.docx]

**Supplemental Table 1. Models Additionally Adjusting for Pre-Pregnancy Weight, Breastfeeding Status, or Physical Activity**

|  | | **Model 1** | | **Model 2** | | | | **Model 3** | | **Model 4** |  |
| --- | --- | --- | --- | --- | --- | --- | --- | --- | --- | --- | --- |
| **Macronutrients** | | **β** | **P-value** | | **β** | | **P-value** | **β** | **P-value** | **β** | **P-value** |
| Protein (g) | -1.09 | | 0.13 | | -0.90 | 0.19 | | -0.93 | 0.16 | -0.86 | 0.18 |
| **Conditionally Essential Amino Acids** | | | | | | | | | |  |  |
| Cysteine (g/d) | | -1.52 | **0.02** | -1.01 | | 0.12 | | -1.33 | **0.04** | -1.40 | **0.02** |
| Arginine (g/d) | | -0.74 | 0.22 | -0.59 | | 0.30 | | -0.56 | 0.35 | -0.68 | 0.23 |
| Glycine (g/d) | | -0.61 | 0.32 | -0.47 | | 0.41 | | -0.46 | 0.43 | -0.59 | 0.28 |
| Proline (g/d) | | -0.99 | 0.23 | -1.24 | | 0.10 | | -0.81 | 0.30 | -0.44 | 0.57 |
| Tyrosine (g/d) | | -1.28 | 0.06 | -1.16 | | 0.07 | | -1.14 | 0.09 | -0.98 | 0.13 |
| **Essential Amino Acids** | |  |  |  | |  | |  |  |  |  |
| Phenylalanine (g/d) | | -1.46 | **0.04** | -1.20 | | 0.08 | | -1.25 | 0.07 | -1.19 | 0.08 |
| Tryptophan (g/d) | | -1.71 | **0.009** | -1.46 | | 0.02 | | -1.60 | **0.01** | -1.48 | **0.02** |
| Valine (g/d) | | -1.34 | **0.04** | -1.11 | | 0.08 | | -1.21 | 0.06 | -1.07 | 0.09 |
| Isoleucine (g/d) | | -1.26 | **0.045** | -1.00 | | 0.09 | | -1.17 | **0.05** | -1.08 | 0.07 |
| Methionine (g/d) | | -0.84 | 0.18 | -0.68 | | 0.25 | | -0.74 | 0.23 | -0.68 | 0.24 |
| Leucine (g/d) | | -1.03 | 0.13 | -0.87 | | 0.17 | | -0.88 | 0.18 | -0.77 | 0.22 |
| Lysine (g/d) | | -0.80 | 0.18 | -0.64 | | 0.25 | | -0.70 | 0.22 | -0.62 | 0.26 |
| Histidine (g/d) | | -0.98 | 0.12 | -0.83 | | 0.16 | | -0.82 | 0.19 | -0.83 | 0.17 |
| Threonine (g/d) | | -1.13 | 0.07 | -0.93 | | 0.12 | | -1.01 | 0.10 | -0.94 | 0.11 |
| **Non-Essential Amino Acids** | |  |  |  | |  | |  |  |  |  |
| Alanine (g/d) | | -0.67 | 0.27 | -0.49 | | 0.40 | | -0.52 | 0.39 | -0.59 | 0.30 |
| Aspartic Acid (g/d) | | -1.14 | 0.07 | -0.90 | | 0.13 | | -0.99 | 0.11 | -0.97 | 0.10 |
| Glutamic Acid (g/d) | | -1.33 | 0.1 | -1.30 | | 0.08 | | -1.14 | 0.14 | -1.01 | 0.18 |
| Serine (g/d) | | -1.18 | 0.09 | -0.88 | | 0.18 | | -0.95 | 0.17 | -0.85 | 0.19 |

**Supplemental Table 1.** Table shows the beta and corresponding p-values before and after including additional covariates, such as pre-pregnancy weight and change in breast feeding status. Reported beta represent one-standard deviation (SD) increase in protein (SD = 17.37 g), cysteine (SD = 0.24 g), proline (SD = 1.09 g), phenylalanine (SD = 0.76 g), tryptophan (SD = 0.21 g), valine (SD = 0.91 g), isoleucine (SD = 0.83 g), arginine (SD = 1.06 g), methionine (SD = 0.46 g), leucine (SD=1.43 g), lysine (SD = 1.4 g), histidine (SD = 0.52 g), threonine (SD = 0.73 g), alanine (SD = 0.94 g), aspartic acid (SD = 1.61 g), glycine (SD = 0.80 g), glutamic acid (SD = 3.17 g), serine (SD = 0.81 g), and tyrosine (SD = 0.64 g). Three models were examined. **Model 1:** 1-Month Age + Height + Total Energy Intake, **Model 2:** 1-Month Age + Height + Total Energy Intake + Pre-pregnancy weight, **Model 3:** 1-Month Age + Height + Total Energy Intake + Change in Breast Feeding Status, and **Model 4:** 1-Month Age + Height + Total Energy Intake + Average Physical Activity.
