# Supplementary material for: Specific amino acids but not total protein attenuate postpartum weight gain among Hispanic women from Southern California
Source: Food Sci Nutr. 2021 Feb 13;9(4):1842–50. doi: 10.1002/fsn3.2085 (PMC8020954; doi:10.1002/fsn3.2085)
Supplement: Supplementary file 3 — Table S2 [file FSN3-9-1842-s003.docx]

**Supplemental Table 2. Correlations between Dietary Protein and Amino Acids Associated with Change in Maternal Weight from 1- to 6-Months Postpartum**

|  | **Protein**  **(g/d)** | **Phenylalanine (g/d)** | **Tryptophan (g/d)** | **Valine (g/d)** | **Isoleucine (g/d)** | **Cysteine (g/d)** |
| --- | --- | --- | --- | --- | --- | --- |
| **Protein (g/d)** | 1.0 |  |  |  |  |  |
| **Phenylalanine (g/d)** | 0.99 | 1.0 |  |  |  |  |
| **Tryptophan (g/d)** | 0.97 | 0.97 | 1.0 |  |  |  |
| **Valine (g/d)** | 0.98 | 0.99 | 0.97 | 1.0 |  |  |
| **Isoleucine (g/d)** | 0.98 | 0.98 | 0.97 | 0.99 | 1.0 |  |
| **Cysteine (g/d)** | 0.94 | 0.94 | 0.92 | 0.93 | 0.94 | 1.0 |

**Supplemental Table 2.** Table presents Pearson correlations between dietary protein and the five dietary amino acids that were significantly associated with postpartum weight loss. All correlations were significant with a p-value of < 2.2e-16.
